# Malcolm Faulk, BSc, MB BS, MPhil, FRCP, FRCPsych

**DOI:** 10.1192/bjb.2025.10117

**Published:** 2026-02

**Authors:** Pamela J. Taylor

Formerly Consultant Forensic Psychiatrist, Wessex Medium Security Hospital Service, Hampshire, UK, and Medical Member of Her Majesty’s Inspectorate of Prisons, London, UK

**Figure:**
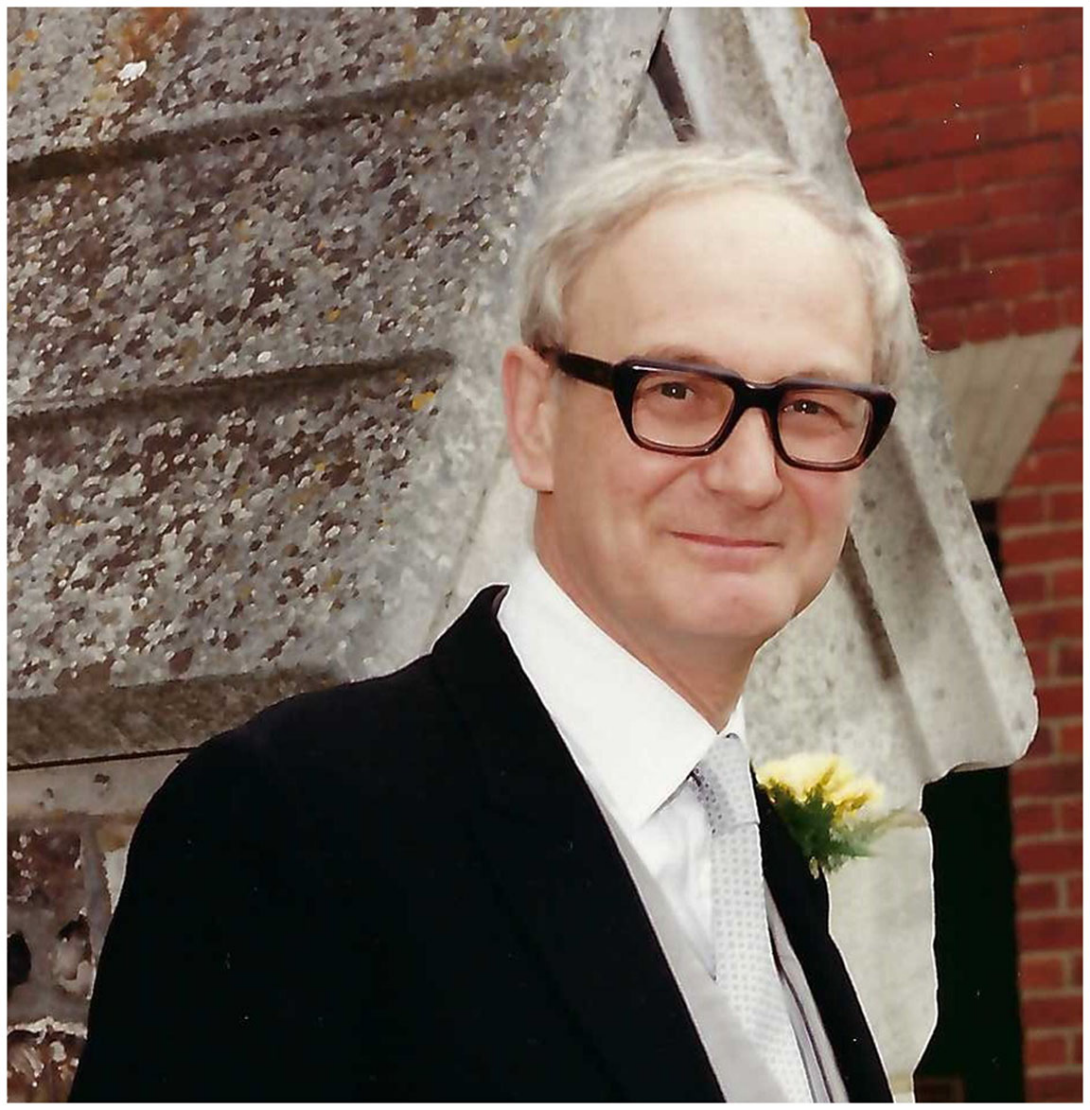

Malcolm Faulk, who died on 13 December 2024, was a pioneer in secure forensic mental health service development. He was a fine clinician, a service visionary and manager, and a writer. Malcolm’s 1988 textbook *Basic Forensic Psychiatry* ^1^ became a standard text for trainees across relevant clinical disciplines and ran to three editions.

Most psychiatrists in the UK have an image of the 1975 Butler Report as the key stimulus for modern forensic psychiatry – and, coupled with earmarked funding, it did indeed ensure developments country-wide. The pathway, however, started earlier, perhaps with the 1957 Percy Commission, paving the way for the Mental Health Act 1959. This allowed liberalisation of most aspects of psychiatric treatment while also embracing a legal category of ‘psychopathic disorder’. The Emery Report and Glancy Report followed, in 1961 and 1974 respectively, highlighting gaps in healthcare provision between high-security hospitals on the one hand and wholly open services on the other. The development of regional secure units was proposed and accepted by the then Department of Health and Social Security. In 1971, Malcolm was one of the first to be appointed jointly between a Regional Health Authority and the Prison Service to develop a new service, ahead of Butler.

On starting to plan this service, for Knowle Hospital, Hampshire, UK, Malcolm immediately experienced the opposition familiar to all such developers – then and later. Already established hospital-based psychiatrists and nurses, fearing that their campus would be filled with dangerous patients and reintroduction of repressive regimes, tried to block the development. The Prison Medical Service, then a parallel Home Office run service, also resisted; Malcolm suggested they saw such developments as a threat to their authority.

Finally, in 1977, Malcolm overcame all obstacles to open the third interim secure unit in the UK (after Rainhill, Merseyside and Prestwich, Manchester) – the 14-bed Ravenswood House at Knowle Hospital. His experience from all this planning, persuasion and implementation was invaluable not only in the design of the later and larger developments at Knowle (1985, 1990) but also in the growth of other such units country-wide. Using the least repressive measures compatible with the necessary security, the so-called Wessex service was particularly successful at integrating with both general and open psychiatric services on the one hand and ‘special’ high-security hospitals on the other, as well as prisons, probation and the courts. A community service followed for the few patients who continued to require specialist forensic mental healthcare to live safely after discharge.

Malcolm was born in Leeds, Yorkshire, on 20 October 1936, the eldest of three. His father, Henry Faulk, was a child of European immigrants who became a schoolteacher (as Lt Colonel in the Intelligence Corps, he was awarded the OBE for his work leading the de-Nazification of over 400 000 German Prisoners of War in Britain). His Mancunian mother, Lena Friedman, was of Russian heritage. All this perhaps fed into Malcolm’s own later creative, caring approaches towards marginalised people. From Queen Elizabeth’s Grammar School, Wakefield, UK, Malcolm entered University College Hospital (UCH) Medical School, London, UK (1955–1962). There, albeit with psychiatry in mind, he completed an intercalated BSc in Comparative Anatomy before qualifying in medicine; Membership of the Royal College of Physicians (MRCP) followed in 1966.

Malcolm married Barbara Heller, a fellow UCH medical student. She was also the child of talented refugees from the European mainland – Hans and Josephine Heller, the former a chemistry and medical graduate who eventually became the first Professor of Pharmacology in Bristol University. Barbara worked in community childcare, forgoing a career in paediatrics in order to raise her family.

Malcolm’s post-graduate psychiatric training was at the Maudsley Hospital, London, UK, 1966–1972, resulting in his Royal College of Psychiatrists’ membership, later fellowship. There he encountered Dr Peter Scott and Professor Trevor Gibbens – the only two designated forensic psychiatrists in Britain at the time – and developed his own forensic psychiatry skills.

Malcolm was a medical member of the Mental Health Review Tribunal 1975–2008 and, after retiring from the National Health Service in 1992, was appointed Medical Member of Her Majesty’s Inspectorate of Prisons, first under Chief Inspector of Prisons Judge (later Sir) Stephen Tumim and then General Sir (later Lord) David Ramsbotham. He retired from the Inspectorate in 1996 to care for his wife, Barbara, when she became terminally ill.

After Barbara’s death in 1999, Malcolm remarried, in 2001, and is survived by his second wife, Chantal Thomas, a French artist. His two children by his first marriage, Matthew and Harriet, and his four grandchildren thrive.

Malcolm’s lifelong hobbies included fishing, watercolour painting, sketching, photography and trying hard to master a sport or musical instrument. He self-published a children’s adventure story that he both wrote and illustrated: *The Illustrated Adventures of Jolyon the Unfortunate Medieval Knight*.^2^
